# Choline and Betaine Intake and Colorectal Cancer Risk in Chinese Population: A Case-Control Study

**DOI:** 10.1371/journal.pone.0118661

**Published:** 2015-03-18

**Authors:** Min-Shan Lu, Yu-Jing Fang, Zhi-Zhong Pan, Xiao Zhong, Mei-Chun Zheng, Yu-Ming Chen, Cai-Xia Zhang

**Affiliations:** 1 Department of Medical Statistics and Epidemiology, School of Public Health, Sun Yat-sen University, Guangzhou, China; 2 Sun Yat-sen University Cancer Center, State Key Laboratory of Oncology in South China, Collaborative Innovation Center for Cancer Medicine, Guangzhou, China; 3 Department of Colorectal Surgery, Sun Yat-sen University Cancer Center, Guangzhou, China; Geisel School of Medicine at Dartmouth College, UNITED STATES

## Abstract

**Background:**

Few studies have examined the association of choline and betaine intake with colorectal cancer risk, although they might play an important role in colorectal cancer development because of their role as methyl donors. The aim of this study was to examine the relationship between consumption of choline and betaine and colorectal cancer risk in a Chinese population.

**Methodology/Principal Findings:**

A case-control study was conducted between July 2010 and December 2013 in Guangzhou, China. Eight hundred and ninety consecutively recruited colorectal cancer cases were frequency matched to 890 controls by age (5-year interval) and sex. Dietary information was assessed with a validated food frequency questionnaire by face-to-face interviews. The logistic regression model was used to estimate multivariate odds ratios (ORs) and 95% confidence intervals (CIs). Total choline intake was inversely associated with colorectal cancer risk after adjustment for various lifestyle and dietary factors. The multivariate-adjusted OR was 0.54 (95%CI = 0.37-0.80, Ptrend <0.01) comparing the highest with the lowest quartile. No significant associations were observed for betaine or total choline+betaine intakes. For choline-containing compounds, lower colorectal cancer risk was associated with higher intakes of choline from phosphatidylcholine, glycerophosphocholine and sphingomyelin but not for free choline and phosphocholine. The inverse association of total choline intake with colorectal cancer risk was observed in both men and women, colon and rectal cancer. These inverse associations were not modified by folate intake.

**Conclusions:**

These results indicate that high intake of total choline is associated with a lower risk of colorectal cancer.

## Introduction

Choline and betaine are widely distributed in plant and animal tissues. Main food sources of choline are eggs, beef liver, chicken liver, wheat germ, bacon, dried soybeans and pork [1]. Food items with the highest content of betaine are wheat bran, wheat germ, spinach, shrimps and wheat bread [1]. Choline is an essential human nutrient [2]. Like folate, it functions as a methyl donor to participate in methyl group metabolism. Choline is oxidized to betaine, which can donate a methyl group to homocysteine to form methionine [3]. Methionine is a precursor to S-adenosylmethionine (SAM)-an important methyl donor in human body. DNA methylation depends upon the availability of methyl groups from SAM and is closely related to the development of several tumor types, including colorectal cancer [4, 5]. Disruption of DNA methylation and impaired DNA repair due to deficiency of methyl donors (folate, choline, betaine and methionine) in one-carbon metabolism are thought to be the main underlying mechanism for carcinogenesis [6].

Some epidemiologic studies have evaluated the association of dietary choline and betaine intakes with some types of cancers [7–14]. However, the results are far from conclusive. The only study thus far to examine the relationship between dietary choline and betaine and colorectal cancer risk is the Health Professionals Follow-up Study conducted in the United States [15]. This study found no significant association between choline and betaine intake and colorectal cancer risk in men. Therefore, more studies in independent populations are warranted to clarify this association.

We hypothesized that higher intake of choline and betaine would be associated with a decreased risk of colorectal cancer. To evaluate this hypothesis, we aimed to examine whether dietary choline and betaine intakes were associated with the risk of colorectal cancer among Chinese population. We also examined whether the associations between choline and betaine intake and colorectal cancer risk were modified by folate intake.

## Methods

### Ethics Statement

This study was approved by the Ethical Committee of School of Public Health of Sun Yat-sen University. All participants in this study provided the written informed consent form prior to the interview.

### Study subjects

This is an ongoing case-control study beginning in July 2010. The selection of cases and controls has been described in detail previously [16]. Briefly, case subjects were consecutively recruited among inpatients admitted in Sun Yat-sen University Cancer Center, Guangzhou, China. To be eligible for the study, cases were required to be incident, histologically confirmed colorectal cancer patients diagnosed no more than 3 months before the interview, aged 30 to 75 years and natives of the Guangdong province or having lived in Guangdong for at least 5 years. Patients were excluded if they had a history of other cancers. Familial adenomatous polyposis and hereditary nonpolyposis colorectal cancer were also excluded in our study. Between July 2010 and December 2013, a total of 982 eligible cases were identified and 890 were successfully interviewed, with a response rate of 90.6%.

Controls were frequency matched to cases by 5-year age group and sex. Eligibility criteria for controls were the same as described for the cases except that they had no history of colorectal cancer. Two control groups were used in this case-control study. The first control group was recruited from the inpatients admitted to three affiliated hospitals of Sun Yat-sen University during the same period as the cases with the following diseases: glaucoma, cataract, keratitis/keratohelcosis/optic neuritis/ pterygium/ dacryocystitis/ ocular trauma/Koyanagi-harada’s syndrome, tympanitis/ sudden deafness, rhinopolyp/sinusitis/deviation of nasal septum, vocal cords cyst/vocal nodules/vocal polyp, varicose veins, tonsillitis/vestibular neuritis/facioplegia/ pigmented naevus/vertigo/ pigmented naevus. In total, 588 hospital-derived controls were identified and invited to participate in the study and 524 were successfully interviewed, yielding a participation rate of 89.1%. The second control group was obtained from the residents with the same community as cases through a variety of strategies such as advertisements, written invitations, or referrals. Totally, 366 community-derived controls were interviewed.

### Data collection

A structured questionnaire was used to collect information by trained interviewers through face-to-face interview. The collected information included the socio-demographic factors, body weight and height, lifestyle factors (e.g., active and passive smoking, alcohol intake, and physical activity), and family history of cancer prior to diagnosis for colorectal cancer patients or interview time for controls. Relevant medical information, medical diagnosis, and histological findings were abstracted from the medical records. Menstrual and reproductive history was also obtained in women. Body mass index (BMI) was calculated by dividing weight (kg) by height squared (m^2^). In this study, regular smokers were defined as someone smoking at least one cigarette a day for more than six consecutive months. Passive smoking meant to be exposed to others’ tobacco smoke for at least 5 minutes per day in previous year. Regular drinking was defined as drinking alcohol at least once per week over the past year. A positive family history with cancer was defined as a self- reported history of cancer in first- or second- degree relatives.

### Dietary intake assessment

Dietary intake was evaluated by a face-to-face interview using an 81-item food frequency questionnaire (FFQ). The referent period for the interview was one year prior to diagnosis for colorectal cancer patients or interview time for controls. For each food item, 5 possible frequencies (never, per year, per month, per week, and per day) and 1 quantitative (amounts) response were available. Food pictures with usual portion size were provided to help participants with quantification of intake. Participants were asked to report how frequently they consumed each food as the number of times per day, per week, per month, per year or never, and the average amount of food eaten each time. For seasonal foods, participants were asked to report how many months they consumed for each food in the previous year. A commonly used portion size was specified for each food (e.g. bowl, slice, glass, or unit, such as one apple or banana). For vegetables and animal foods, a Liang (1 Liang = 50 grams), a common weight measure familiar to the study subjects, was used to estimate the usual portion size. Daily dietary nutrient intakes including choline, betaine and other nutrients were calculated based on the Chinese Food Composition Table [17] and values published by Zeisel *et al* [1, 18]. Total dietary intakes of energy, the five choline-containing compounds (phosphatidylcholine, glycerophosphocholine, phosphocholine, sphingomyelin and free choline), and betaine were calculated by summing the product of the frequency of consumption, usual portion consumed, and nutrient content of each food item. Total choline intake was calculated by summing choline from phosphatidylcholine, glycerophosphocholine, phosphocholine, sphingomyelin and free choline.

As part of the study, we also collected information on whether study subjects had changed their appetite and dietary habits in the past one year before the interview and whether they used vitamin supplements. Study subjects were considered as having significant dietary changes if they reported “Yes” to both of the following questions: “Compared with the previous years, have you changed your appetite in the past one year?” and “Compared with the previous years, have you changed your dietary habits in the past one year?”. Study subjects were considered as users of vitamin supplements if they reported “Yes” to question: “In the past one year did you take vitamin supplements for more than 3 months?”

The validity and reproducibility of the FFQ with 6 3-day dietary records have been confirmed among women who lived in the same region [7, 19]. The correlation coefficients comparing the second FFQ and 18-day dietary records were 0.34 for total choline, 0.26 for betaine, 0.48 for glycerophosphocholine, 0.44 for phosphocholine, 0.23 for phosphatidylcholine, and 0.36 for sphingomyelin and free choline, respectively. The correlation coefficients between the 2 FFQs of total choline, betaine, phosphatidylcholine, glycerophosphocholine, phosphocholine, sphingomyelin, and free choline were 0.59, 0.44, 0.56, 0.67, 0.64, 0.54, and 0.58, respectively.

### Statistical analysis

SPSS 13.0 (SPSS Inc., Chicago, Illinois, USA) was used to conduct all data analysis. The chi-square test was used to test the difference between cases and controls in terms of categorical variables and *t*-test or Wilcoxon rank-sum test was used for continuous variables. Participants were categorized into quartiles on the basis of the distribution of each dietary factor among controls for men and women separately. Unconditional logistic regression models were used to assess the odds ratios (ORs) and 95% confidence intervals (CIs) for the association of choline and betaine consumption with colorectal cancer risk, using the lowest quartile as reference. Multivariate-adjusted models included age (continuous), marital status (married/other), education (primary school or blow, secondary school, high school, college or above), income level (<2,000/2,001–5,000/5,001–8,000/>8,001), occupation (white collar worker, blue collar worker, farmer or other), family history of cancer (yes/no), smoking status (current/never or past), passive smoking (yes/no), alcohol drinking (yes/no), physical activity (less active, moderate active, more active), and BMI (continuous), which were selected based on the literatures and comparison of the characteristics between case and control subjects. Folate intake was also treated as a confounder because it was significantly correlated with total choline and betaine. The multivariate analyses in women were also adjusted for menopausal status. All nutrients were adjusted for total energy intake by using the residual method [20]. Tests for trend were conducted by entering the categorical variables as continuous variables in multiple regression models.

Since both folate and choline (through betaine) can donate a methyl group to homocysteine and are involved in methyl-group metabolism, analysis stratified by folate intake values below and above the median (<234 *vs* ≥234 μg/d) intake was conducted to evaluate the potential modifying effect on choline and betaine intake and colorectal cancer risk. Stratified analyses according to sex, BMI, smoking status and alcohol drinking were also conducted. The interaction was evaluated by multiplicative models by including the product term in multivariate logistic regression. Subgroup analyses by cancer sites (colon or rectal cancer) and sources of controls (hospital-derived or community-derived) were conducted for the association between dietary choline and betaine intake and colorectal cancer risk. The sensitivity of results excluding study subjects with dietary changes and with vitamin supplements was assessed. In this study, all statistical tests were based on two-tailed probability values with *P* values of ≤0.05 interpreted as being statistically significant.

## Results

Of the 890 cases (495 men and 395 women), 533 were classified as colon cancer (312 men and 221 women), and 357 were classified as rectal cancer (183 men and 174 women).

Table 1 shows the demographic characteristics of study subjects and selected colorectal cancer risk factors. Characteristics of hospital-derived controls and community-derived controls are also shown in Table 1. Compared to the control subjects, cases tended to have a higher household income and a lower educational level, to be less physical active, were more likely to suffer passive smoking exposure and to have family history of cancer. No significant differences between cases and controls were observed for age, residence, occupation, marital status, regular smoking, regular drinking, and BMI. There were significant differences between two control groups in some characteristics and selected colorectal cancer risk factors.

**Table 1:** Demographic and selected risk factors of colorectal cancer cases and controls in Chinese population.

	Cases	Controls	*P-*value	Community-	Hospital-	*P-*value
	(n = 890)	(n = 890)	between	Derived	Derived	Between
			cases	controls	controls	control
			and controls	(*n* = 366)	(*n* = 524)	groups
Age, yr, (mean±SD)	56.6 ± 10.3	56.5 ± 10.0	0.83	63.3 ± 6.2	51.8 ± 9.4	<0.01
Sex (n, %)			1			<0.01
Men	495 (55.6)	495 (55.6)		262 (71.6)	233 (44.5)	
Women	395 (44.4)	395 (44.4)		104 (28.4)	291 (55.5)	
Marital status (n, %)			0.29			0.15
Married	849 (95.4)	839 (94.3)		340 (92.9)	499 (95.2)	
Unmarried/divorces/widowed	41 (4.6)	51 (5.7)		26 (7.1)	25 (4.8)	
Residence (n, %)			1			0.06
Urban	602 (67.6)	602 (67.6)		261 (71.3)	341 (65.1)	
Rural	288 (32.4)	288 (32.4)		105 (28.7)	183 (34.9)	
Educational Level (n, %)			<0.01			<0.01
Primary school or blow	279 (31.3)	207 (23.3)		63 (17.2)	144 (27.5)	
Secondary school	229 (25.7)	238 (26.7)		76 (20.8)	162 (30.9)	
High school	221 (24.8)	243 (27.3)		123 (33.6)	120 (22.9)	
College or Above	161 (18.1)	202 (22.7)		104 (28.4)	98 (18.7)	
Occupation (n, %)			0.11			<0.01
Administrator/other white collar worker	119 (13.4)	137 (15.4)		20 (5.5)	117 (22.4)	
Blue collar worker	179 (20.1	203 (22.8)		53 (14.5)	150 (28.7)	
Farmer/other	592 (66.5)	549 (61.8)		293 (80.1)	256 (48.9)	
Income (Yuan/month) (n, %)			<0.01			<0.01
<2,000	135 (15.2)	139 (15.7)		38 (10.4)	101 (19.3)	
2,001–5,000	282 (31.7)	309 (34.8)		164 (45.1)	145 (27.7)	
5,001–8,000	238 (26.7)	287 (32.3)		129 (35.4)	158 (30.2)	
>8,001	235 (26.4)	150 (16.9)		30 (8.2)	120 (22.9)	
BMI (mean±SD)	22.9 ± 3.5	23.2 ± 3.1	0.05	23.7 ± 3.0	22.9 ± 3.1	0.21
Regular smoker (n, %)	238 (26.7)	272 (30.6)	0.08	126 (34.4)	146 (27.9)	0.04
Passive smoking (n, %)	548 (61.6)	414 (46.6)	<0.01	72 (19.7)	342 (65.3)	<0.01
Regular drinker (n, %)	145 (16.3)	128 (14.4)	0.29	50 (13.7)	78 (14.9)	0.63
Family history of cancer (n, %)	133 (14.9)	80 (9.0)	<0.01	31 (8.5)	49 (9.4)	0.72
Physical activity (n, %)			<0.01			<0.01
Less active	473 (53.1)	458 (51.6)		145 (39.8)	313 (59.7)	
Moderate active	296 (33.3)	223 (25.1)		133 (36.5)	90 (17.2)	
More active	121 (13.6)	207 (23.3)		86 (23.6)	121 (23.1)	
Menopausal status (n, %) ^a^			0.35			<0.01
Premenopausal	114 (28.9)	127 (32.2)		4 (3.8)	123 (42.3)	
Postmenopausal	281 (71.1)	268 (67.8)		100 (96.2)	168 (57.7)	

^a^ Among women subgroup.

Major food sources for total choline, betaine, and five choline-containing compounds are shown in Table 2. Animal-based foods including eggs, chicken, whole milk, and vegetables such as broccoli and spinach were the main food sources of total choline. Vegetables such as spinach and potatoes and grain products were the main food sources of betaine. Approximately 60% of choline was consumed in the form of phosphatidylcholine, followed by free choline (22%), glycerophosphocholine (9%), phosphocholine (8%), and sphingomyelin (3%).

**Table 2:** Top five food sources of total choline, choline-containing compounds, and betaine among control subjects.

Food sources	Proportion	Food sources	Proportion
Folate		Free choline (22% of choline)	
Rice	18.44	Broccoli	24.61
Chinese cabbage	10.14	Chinese cabbage	11.31
Eggs	9.15	Pasta	7.45
Pasta	5.28	Whole milk	4.28
Spinach	5.07	Potatoes	3.24
Total choline		Glycerophosphocholine (9% of choline)	
Eggs	28.64	Whole milk	22.03
Broccoli	17.25	Rice	12.53
Chicken	8.72	Yoghourt	8.84
Whole milk	3.68	Banana	7.32
Spinach	3.25	Broccoli	6.47
Betaine		Phosphocholine (8% of choline)	
Spinach	60.06	Broccoli	48.05
Pasta	23.94	Chinese cabbage	7.56
White bread	6.30	Chicken	6.29
Wheat bread	4.22	Whole milk	5.71
Potatoes	2.63	Potatoes	4.48
Phosphatidylcholine (58% of choline)		Sphingomyelin (3% of choline)	
Eggs	47.06	Chicken	47.16
Broccoli	15.63	Eggs	37.48
Chicken	10.21	Whole milk	5.11
Spinach	4.63	Yoghourt	2.84
Potatoes	2.41	White bread	2.64

Among control subjects, the energy-adjusted median intakes of total choline and betaine were 158.2 mg/d and 230.6 mg/d, respectively. Compared to control subjects, cases had lower intakes of total choline, the five choline-containing compounds and betaine (Table 3).

**Table 3:** Comparison of energy, total choline, individual choline-containing compounds and betaine between colorectal cancer cases and controls.

	Cases (n = 890)			Controls (n = 890)			
	Mean	SD	Median (25th, 75th)	Mean	SD	Median (25th, 75th)	*P* value
Energy (kJ/day)	6592	2057	6354 (5095, 7787)	7336	2346	7002 (5668, 8573)	<0.01
Total choline (mg/day) ^a^	142.0	56.47	132.7 (99.68, 176.4)	164.9	63.89	158.2 (119.5, 201.7)	<0.01
Choline from phosphatidylcholine (mg/day) ^a^	82.06	39.09	75.45 (52.92, 104.6)	95.67	46.96	89.46 (64.38, 119.8)	<0.01
Free choline (mg/day) ^a^	32.74	11.88	31.10 (24.10, 39.61)	36.11	12.11	34.93 (27.31, 42.84)	<0.01
Choline from glycerophosphocholine (mg/day) ^a^	10.37	5.92	8.51 (6.80, 11.56)	14.13	8.59	11.04 (7.98, 18.06)	<0.01
Choline from phosphocholine (mg/day) ^a^	12.92	7.09	11.56 (7.77, 16.93)	14.02	6.82	12.81 (9.09, 17.58)	<0.01
Choline from sphingomyelin (mg/day) ^a^	3.97	2.48	3.53 (2.27, 5.30)	5.25	3.25	4.76 (2.94, 6.93)	<0.01
Betaine (mg/day) ^a^	245.2	191.8	206.1 (116.7, 323.1)	266.1	173.5	230.6 (150.5, 338.9)	<0.01
Total choline+betaine (mg/day) ^a^	424.2	302.9	360.4 (224.7, 535.5)	463.7	275.0	407.2 (289.0, 559.7)	<0.01
Folate (ug/day) ^a^	215.4	53.02	210.3 (178.5, 244.8)	240.3	64.20	233.6 (195.5, 277.6)	<0.01
Beans(g/day)	28.89	33.84	17.86 (8.02, 36.68)	39.75	55.38	20.42 (8.32, 50.55)	<0.01
Red meat(g/day)	117.2	77.38	102.4 (68.57, 146.4)	108.7	75.55	93.30 (58.03, 141.7)	<0.01
Fish(g/day)	54.95	55.93	39.17 (19.82, 71.43)	135.3	133.6	86.31 (35.24, 198.4)	<0.01

^a^ Intakes of total choline, individual choline-containing compounds and betaine were adjusted for the daily energy intake using the residual method.

Wilcoxon rank-sum test comparing the median consumption levels between cases and controls.

Total choline, betaine, and the five main choline-containing compounds were all significantly correlated, except for choline from sphingomyelin and betaine. The Spearman’s correlation coefficients ranged from 0.222 to 0.945 (Table 4).

**Table 4:** Correlation coefficients between energy-adjusted folate, betaine, choline and individual choline-containing compounds in controls (two-tailed Spearman).

	Folate	Total choline	Betaine	Choline from phosphatidylcholine	Free choline	Choline from glycerophosphocholine	Choline from phosphocholine	Choline from sphingomyelin
Folate	1.000	0.654**	0.418**	0.593**	0.661**	0.431**	0.435**	0.381**
Total choline		1.000	0.273**	0.945**	0.740**	0.621**	0.674**	0.716**
Betaine			1.000	0.222**	0.392**	0.198**	0.275**	0.084*
Choline from phosphatidylcholine				1.000	0.538**	0.429**	0.497**	0.742**
Free choline					1.000	0.640**	0.806**	0.312**
Choline from glycerophosphocholine						1.000	0.502**	0.457**
Choline from phosphocholine							1.000	0.255**
Choline from sphingomyelin								1.000

** Correlations are significant (*p*<0.01).

* Correlations are significant (*p*<0.05)

Table 5 presents the ORs and 95% CIs for colorectal cancer risk according to quartiles of total choline and betaine consumptions. After adjusting for socio-demographic and lifestyle factors, the ORs for colorectal cancer risk in the highest quartile of intakes, compared with the lowest quartile, were 0.31 (95%CI = 0.23–0.42) for total choline, 0.63 (95%CI = 0.50–0.87) for betaine, and 0.59 (95%CI = 0.44–0.77) for total choline+betaine intake. This inverse association persisted after further adjustment for non-choline or betaine sources of foods, including red meat, fish and beans. After further adjustment for folate, the inverse associations of betaine, total choline + betaine with colorectal cancer risk attenuated, and only high intake of total choline remained associated with a reduced risk of colorectal cancer, with adjusted OR (95%CI) of 0.54 (0.37–0.80) comparing the highest with the lowest quartile of total choline intake.

**Table 5:** Odds ratios (ORs) and 95% confidence intervals (95%CIs) of colorectal cancer according to quartiles of choline and betaine intake.

	Q1	Q2	Q3	Q4	*P*trend
Total choline					
No. Cases/Controls	360/222	220/223	188/223	122/222	
Crude OR (95%CI)	1.00	0.61 (0.47–0.78)	0.52 (0.40–0.67)	0.34 (0.26–0.45)	<0.01
Adjusted OR (95%CI) ^a^	1.00	0.57 (0.44–0.74)	0.48 (0.36–0.63)	0.31 (0.23–0.42)	<0.01
Adjusted OR (95%CI) ^b^	1.00	0.51 (0.38–0.69)	0.43 (0.31–0.58)	0.29 (0.20–0.40)	<0.01
Adjusted OR (95%CI) ^d^	1.00	0.61 (0.45–0.83)	0.63 (0.45–0.88)	0.54 (0.37–0.80)	<0.01
Betaine					
No. Cases/Controls	296/223	204/221	197/224	193/222	
Crude OR (95%CI)	1.00	0.70 (0.54–0.90)	0.66 (0.51–0.86)	0.65 (0.51–0.85)	<0.01
Adjusted OR (95%CI) ^a^	1.00	0.68 (0.51–0.89)	0.66 (0.50–0.87)	0.63 (0.50–0.87)	<0.01
Adjusted OR (95%CI) ^c^	1.00	0.63 (0.46–0.85)	0.55 (0.41–0.76)	0.58 (0.42–0.79)	<0.01
Adjusted OR (95%CI) ^e^	1.00	0.71 (0.52–0.97)	0.74 (0.53–1.02)	0.92 (0.65–1.29)	0.56
Total choline+betaine					
No. Cases/Controls	314/222	205/223	184/223	187/222	
Crude OR (95%CI)	1.00	0.65 (0.50–0.84)	0.58 (0.45–0.76)	0.60 (0.46–0.77)	<0.01
Adjusted OR (95%CI) ^a^	1.00	0.65 (0.50–0.85)	0.58 (0.44–0.77)	0.59 (0.44–0.77)	<0.01
Adjusted OR (95%CI) ^b^	1.00	0.59 (0.44–0.80)	0.49 (0.36–0.66)	0.54 (0.39–0.74)	<0.01
Adjusted OR (95%CI) ^d^	1.00	0.72 (0.52–0.98)	0.69 (0.50–0.95)	0.91 (0.65–1.28)	0.46

^a^ Odds ratio was adjusted for age (continuous), sex (men/women), residence (urban/rural), marital status (married/other), education (primary school or blow/secondary school/high school/college or above), income level (<2,000/2,001–5,000/5,001–8,000/>8,001), occupation (white collar worker/blue collar worker/farmer or other), family history of cancer (yes/no), smoking status (current/never or past), passive smoking (yes/no), alcohol drinking (yes/no), degree of physical activity (less active/moderate active/more active), BMI (continuous).

^b^ Odds ratio was adjusted for the various above confounders and red meat (continuous), fish (continuous).

^c^ Odds ratio was adjusted for the various above confounders and red meat (continuous), fish (continuous), beans (continuous).

^d^ Odds ratio was adjusted for the various above confounders in ^b^ and folate intake (continuous).

^e^ Odds ratio was adjusted for the various above confounders in ^c^ and folate intake (continuous).

For the five compounds derived from choline, higher intakes of choline from phosphatidylcholine, glycerophosphocholine and sphingomyelin were found to be inversely associated with colorectal cancer risk. After adjustment for various dietary and nondietary confounders, participants in the highest quartile of intakes had multivariate ORs (95%CI) of 0.81 (0.59–1.12) for phosphatidylcholine, 0.30 (0.21–0.43) for glycerophosphocholine, and 0.35 (0.25–0.49) for choline from sphingomyelin, compared with those in the lowest quartiles of intakes. However, choline from phosphocholine and free choline were not found to be associated with colorectal cancer risk (Table 6).

**Table 6:** Odds ratios (ORs) and 95% confidence intervals (95%CIs) of colorectal cancer according to quartiles of five main choline-containing compounds intakes.

	Q1	Q2	Q3	Q4	*P*trend
Choline from phosphatidylcholine					
No. Cases/Controls	342/221	231/223	161/224	156/222	
Crude OR (95%CI)	1.00	0.67 (0.52–0.86)	0.46 (0.36–0.60)	0.45 (0.35–0.59)	<0.01
Adjusted OR (95%CI) ^a^	1.00	0.62 (0.47–0.80)	0.42 (0.32–0.56)	0.44 (0.33–0.58)	<0.01
Adjusted OR (95%CI) ^b^	1.00	0.76 (0.58–1.00)	0.59 (0.44–0.80)	0.81 (0.59–1.12)	0.04
Choline from glycerophosphocholine					
No. Cases/Controls	366/221	279/224	160/224	85/221	
Crude OR (95%CI)	1.00	0.75 (0.59–0.96)	0.43 (0.33–0.56)	0.23 (0.17–0.31)	<0.01
Adjusted OR (95%CI) ^a^	1.00	0.69 (0.53–0.89)	0.37 (0.28–0.49)	0.21 (0.15–0.29)	<0.01
Adjusted OR (95%CI) ^b^	1.00	0.81 (0.62–1.06)	0.49 (0.36–0.65)	0.30 (0.21–0.43)	<0.01
Choline from phosphocholine					
No. Cases/Controls	301/222	209/223	181/222	199/223	
Crude OR (95%CI)	1.00	0.69 (0.53–0.89)	0.60 (0.46–0.78)	0.66 (0.51–0.85)	<0.01
Adjusted OR (95%CI) ^a^	1.00	0.70 (0.54–0.92)	0.58 (0.44–0.77)	0.60 (0.46–0.80)	<0.01
Adjusted OR (95%CI) ^b^	1.00	0.87 (0.66–1.15)	0.82 (0.61–1.01)	0.95 (0.70–1.28)	0.60
Choline from sphingomyelin					
No. Cases/Controls	335/223	280/222	184/223	91/222	
Crude OR (95%CI)	1.00	0.84 (0.66–1.07)	0.55 (0.42–0.71)	0.27 (0.20–0.37)	<0.01
Adjusted OR (95%CI) ^a^	1.00	0.83 (0.64–1.08)	0.54 (0.41–0.71)	0.26 (0.19–0.36)	<0.01
Adjusted OR (95%CI) ^b^	1.00	0.97 (0.74–1.28)	0.66 (0.49–0.88)	0.35 (0.25–0.49)	<0.01
Free choline					
No. Cases/Controls	335/223	225/222	170/224	160/221	
Crude OR (95%CI)	1.00	0.67 (0.52–0.87)	0.51 (0.39–0.66)	0.48 (0.37–0.63)	<0.01
Adjusted OR (95%CI) ^a^	1.00	0.63 (0.49–0.83)	0.47 (0.35–0.62)	0.44 (0.33–0.59)	<0.01
Adjusted OR (95%CI) ^b^	1.00	0.78 (0.60–1.03)	0.70 (0.52–0.95)	0.91 (0.65–1.57)	0.29

^a^ Odds ratio was adjusted for age (continuous), sex (men/women), residence (urban/rural), marital status (married/other), education (primary school or blow/secondary school/high school/college or above), income level (<2,000/2,001–5,000/5,001–8,000/>8,001), occupation (white collar worker/blue collar worker/farmer or other), family history of cancer (yes/no), smoking status (current/never or past), passive smoking (yes/no), alcohol drinking (yes/no), degree of physical activity (less active/moderate active/more active), and BMI (continuous).

^b^ Odds ratio was adjusted for the various above confounders and folate intake (continuous).

The inverse association between total choline intake and colorectal cancer risk did not differ appreciably stratified by folate intake. Compared with the lowest quartile, the adjusted OR in the highest quartile of intakes was 0.38 (95%CI = 0.20–0.71, *P*_trend_<0.01) among population with low folate level and 0.43 (95%CI = 0.23–0.79, *P*_trend_<0.01) among those with a high folate intake (*P*_interaction_ = 0.50). However, the inverse association between betaine and colorectal cancer risk disappeared after stratification by folate intake, with the adjusted OR (95%CI) of 0.89 (0.58–1.36, *P*_trend_ = 0.33) and 0.91 (0.56–1.47, *P*_trend_ = 0.81) (*P*_interaction_ = 0.29) (Table 7). Analyses stratified by sex were also conducted. The inverse associations between total choline intake and colorectal cancer risk were observed in both men and women (*P*_interaction_ = 0.78). No interaction of colorectal cancer risk was observed between BMI, smoking status and alcohol intake and total choline intake.

**Table 7:** Odds ratios (ORs) and 95% confidence intervals (95%CIs) of colorectal cancer according to quartiles of total choline and betaine intake stratified by selected variables.

	**Total choline**					**Betaine**				
	Q1	Q2	Q3	Q4	*P*trend	Q1	Q2	Q3	Q4	*P*trend
**Folate intake** ^a^										
Folate <234 μg/day										
No. Cases/Controls	325/194	164/135	97/83	20/33		246/159	156/127	128/102	76/57	
Adjusted OR (95%CI) ^b^	1.00	0.68 (0.50–0.92)	0.68 (0.47–0.98)	0.38 (0.20–0.71)	<0.01	1.00	0.80 (0.58–1.11)	0.80 (0.56–1.13)	0.89 (0.58–1.36)	0.33
Folate≥234 μg/day										
No. Cases/Controls	35/28	56/88	91/140	102/189		50/64	48/94	69/122	117/165	
Adjusted OR (95%CI) ^b^	1.00	0.54 (0.28–1.05)	0.51 (0.27–0.94)	0.43 (0.23–0.79)	<0.01	1.00	0.64 (0.37–1.12)	0.76(0.45–1.28)	0.91 (0.56–1.47)	0.81
*P*_interaction_	0.50					0.29				
**Sex**										
Men										
No. Cases/Controls	201/123	124/125	108/124	62/123		164/124	120/123	99/124	112/124	
Adjusted OR (95%CI) ^c^	1.00	0.67 (0.46–0.99)	0.74 (0.49–1.12)	0.57 (0.35–0.94)	0.04	1.00	0.78 (0.53–1.16)	0.74 (0.49–1.12)	0.99 (0.66–1.51)	0.87
Women										
No. Cases/Controls	159/99	96/98	80/99	60/99		132/99	84/98	98/100	81/98	
Adjusted OR (95%CI) ^d^	1.00	0.65 (0.43–0.99)	0.61 (0.39–0.95)	0.61 (0.37–1.01)	0.03	1.00	0.68 (0.44–1.04)	0.96 (0.63–1.47)	0.86 (0.55–1.35)	0.78
*P*_interaction_	0.78					0.60				

^a^ Intakes of folate were adjusted for the daily energy intake using the residual method.

^b^ Odds ratio was adjusted for age (continuous), sex (men/women), residence (urban/rural), marital status (married/other), education (primary school or blow/secondary school/high school/college or above), income level (<2,000/2,001–5,000/5,001–8,000/>8,001), occupation (white collar worker/blue collar worker/farmer or other), family history of cancer (yes/no), smoking status (current/never or past), passive smoking (yes/no), alcohol drinking (yes/no), degree of physical activity (less active/moderate active/more active), and BMI (continuous).

^c^ Odds ratio was adjusted for the various above confounders except sex and folate intake (continuous).

^d^. Odds ratio was adjusted for the various above confounders and menopausal status in women.

Subgroup analysis by cancer site showed that the inverse associations between total choline intake and colorectal cancer risk were found in both colon and rectal cancers (data not shown). Since different conclusion might be reached when different controls were used, we further conducted subgroup analyses for the choline-colorectal cancer association according to the source of control subjects. An inverse relationship between total choline intake and colorectal cancer risk was observed by using hospital controls and community controls (data not shown). Sensitivity analysis excluding those subjects with dietary changes (29 for cases and 55 for controls) was assessed and it showed no substantial change (data not shown). In our study, 37 (4.2%) cases and 111 (12.5%) controls reported to have vitamin supplements. Excluding study subjects with vitamin supplements did not change the results.

## Discussion

In this case-control study conducted in a Chinese population, we observed an inverse association between total choline intake and colorectal cancer risk. For the individual choline compound, intakes of phosphatidylcholine, glycerophosphocholine and sphingomyelin were also found to be inversely associated with colorectal cancer risk. No statistically significant associations were observed between consumption of betaine, choline from phosphocholine, and free choline and colorectal cancer risk. Similar results were found in subgroups of men and women, colon and rectal cancer, and with hospital and community controls. The protective effect of total choline intake with colorectal cancer was not modified by dietary folate intake.

To our knowledge, so far only the Health Professionals Follow-up Study conducted in the United States has examined the association between choline and betaine intake and colorectal cancer risk [15]. This prospective cohort study suggested that choline and betaine intake had no influence on colorectal cancer risk in men, with the adjusted relative risk (95% CI) of 0.97 (0.79–1.20) for choline intake and 0.94 (0.77–1.16) for betaine intake comparing the highest quartile with the lowest quartile. However, the Nurses’ Health Study examining the relationship between dietary choline and betaine and the risk of distal colorectal adenoma in women showed that increasing choline intake was associated with an elevated risk of colorectal adenoma; the relative risk was 1.45 (95%CI = 1.27–1.67) comparing top quintile with bottom quintile [21]. Inconsistent results were also observed on the relationships between choline and betaine intake and other types of cancers. Four studies evaluated the association of choline and betaine intake wih breast cancer risk and the results remained inconclusive. We previously found a decreased risk of breast cancer risk with choline and betaine intake [7]. A population-based case-control study conducted in Long Island, New York also found an inverse association between choline intake and breast cancer risk [8]. However, the other two studies found no evidence that higher intakes of choline and betaine reduced the risk of breast cancer among premenopausal [9] or postmenopausal women [10]. Some studies have reported that higher intakes of choline and betaine were associated with the reduced risk of lung cancer [11] and nasopharyngeal carcinoma [14], whereas no association was found for epithelial ovarian cancer [12]. Richman *et al* [13] even found an increased risk of prostate cancer in men with high choline intake. The results of the current study were consistent with the findings of Zhang et al [7] and Zeng et al [14] conducted in the same geographic area as our study, showing that increased intakes of total choline may reduce the risk of colorectal cancer.

Choline from phosphatidylcholine and sphingomyelin are fat-soluble choline- containing compounds, and free choline, choline from phosphocholine, and glycerophosphocholine are water-soluble choline-containing compounds. Different choline-containing compounds might have different bioavailability. However, the present study showed that the protective effect was found in both fat-soluble choline-containing compounds (choline from phosphatidylcholine and sphingomyelin) and water-soluble choline-containing compound (glycerophosphocholine). It means that choline from different food sources (animal or plant) did not show different effects on colorectal cancer risk.

Among possible explanations of the inconsistent results of different studies, differences in folate intake between various populations should be taken into account. Both folate and choline (through betaine) are involved in methyl-group metabolism as methyl-group donors. The folate and choline metabolic pathways therefore are closely interrelated. The Health Professionals Follow-up Study mentioned above found that choline and betaine intake was not associated with colorectal cancer risk [15]. Lack of association might be that choline or betaine intakes were not critical in folate-nourished populations. Mean dietary (only from foods) and total folate (from foods and supplements) intakes were 522 and 858μg/day in the Health Professionals Follow-up Study [22]. This indicated that the folate intake level was high in the study population. However, unlike Western populations, most Chinese population consumes unfortified and processed foods. Folate was mainly from natural foods. In the present study, the energy-adjusted median dietary folate intake was 234μg/day among controls and therefore the intake level of folate was not very high. Previous study has shown that choline can be utilized as a methyl donor when folate intake is low [23]. Therefore, relatively lower dietary folate intake in the present study contributed to the detection of the protective effect of choline intake on colorectal cancer risk.

The different intake levels and food sources of choline and betaine in different studies might also explain the different results. The energy-adjusted mean choline and betaine intake in the control group of the present study were 165mg/day and 266 mg/day, which were comparable with another study also conducted in China (203 and 314 mg/day) [14]. Eggs, broccoli, chicken, whole milk, and spinach were the main food sources of total choline, and vegetables and grain products were the main food sources of betaine. However, in the Nurses’ Health Study [21], mean choline intake was 331 mg/day which was about twice as high as in the current study. On the contrary, betaine levels in the current study are relatively high compared to the Nurses’ Health Study (189 mg/day). In the Nurses’ Health Study, animal-based foods, including red meat, eggs, poultry and milk were the main sources of choline; spinach, white bread, cold breakfast cereal, pasta, and dark bread were the main sources of betaine.

Both choline and betaine intake were significantly correlated with folate intake. Further adjustment for folate intake did not appreciably change the relationship between total choline intake and colorectal cancer risk, whereas the inverse association between betaine intake and colorectal cancer risk disappeared. The results showed that the crude OR (95%CI) with colorectal cancer risk was 0.34 (0.26–0.45) for total choline and 0.65 (0.51–0.85) for betaine, respectively. The relatively stronger unadjusted inverse association between total choline and colorectal cancer risk was less likely to be affected by the potential confounders. This might explain the differences after further adjustment for folate intake.

Although the exact mechanism by which high consumption of total choline protects against colorectal cancer risk remains unclear, the protective effect of choline intake on carcinogenesis is biologically reasonable. Choline is a necessary source of methyl groups for methyl group transfer and can be oxidized to betaine to participating in DNA methylation, especially in folate-deficiency populations. Deficiency of methyl donors might lead to the disruption of DNA methylation and impaired DNA repair [6].

### Strengths

The strengths of the present study included the relatively large sample size, the collection of a wide range of potential confounders, assessment of portion size by means of visual aids, the use of the face-to-face interviews, and the consistency of the protective effect of choline-containing compounds derived from animal or plant foods.

### Limitations

This study had some limitations. First, colorectal cancer patients were recruited from only one hospital, Sun Yat-sen University Cancer Center. However, this is the biggest cancer center in the South China. Colorectal cancer patients admitted to this hospital [24] had similar clinical characteristics to patients in other two big hospitals in Guangdong province [25] and those in mainland China [26]. On the other hand, the use of hospital-based controls with conditions potentially related to diet is also of a major concern. To minimize this bias, hospital-derived controls were recruited from several conditions with no apparent association with a dietary cause. Furthermore, two control groups were used in the present study. The same results of different sources of controls indicated that the selection of controls did not affect the results. Therefore, selection bias should not be a serious problem.

Second, recall bias is also of concern in case-control studies. The patients who were aware of their own diagnosis may change their dietary habits consciously. To reduce this bias, an attempt was made to interview the cases as soon as diagnosis was made. Photographs of foods with usual portion size were also used to help participants accurately estimate the food intake. Moreover, the consistency of the inverse association across three choline compounds (choline from phosphatidylcholine, glycerophosphocholine and sphingomyelin) argues against recall bias to some degree, since these compounds come from different types of foods (esp. animal *vs*. plant) that would be mis-reported in different directions.

Third, dietary intake was assessed for one year before diagnosis for colorectal cancer cases or interview for controls. Because there is a long time lag between dietary exposure and the onset of colorectal cancer, dietary changes prior to the 1-year mark would alter the relationship between choline and betaine intake and colorectal cancer risk. However, sensitivity analysis that excluded those cases and controls with substantial changes in dietary habits in the past year revealed similar results as compared with the analyses that included those with substantial changes. Furthermore, adults generally maintain a relatively stable eating habit for a long time [27]. Therefore, the results of this study are unlikely to be greatly affected by potential changes in eating habits.

Fourth, FFQ was used to collect information of dietary intake in the present study. Although it has been validated among women who lived in the same region [7, 19], FFQ was not validated among men. Although the correlation coefficients for choline and betaine comparing FFQ with 18-day dietary records are comparable to the published data, they are relatively low. This showed that these nutrients appear to be some of the worst measured and measurement errors inherent in such dietary assessment apply to our study. However, this misclassification is most likely non-differential and thus does not explain the inverse association observed in our study.

Fifth, although we have adjusted for several confounding factors, some residual confounding may result from misclassification of those variables and confounding by unmeasured variables.

## Conclusions

In conclusion, the results of this study support the hypothesis that consumption of choline, betaine, and choline-containing compounds was inversely associated with colorectal cancer risk. Further studies, especially prospective cohort studies from other populations are necessary to confirm this association.
